# PP11 EVTESA: Transforming Social Security In The Dominican Republic

**DOI:** 10.1017/S0266462325102316

**Published:** 2025-12-29

**Authors:** Madeline Martinez, Yasmiry Mejía

## Abstract

**Introduction:**

The development and implementation of the health technology assessment process by the Superintendence of Health and Labor Risks (SISALRIL) optimizes the inclusion of technologies in the Basic Health Plan, based on scientific evidence. This process (known as EVTESA) strengthens the social security system in the Dominican Republic by promoting informed decision-making, transparency, and spending efficiency; ensuring access to safe and effective technologies; and addressing historical challenges in the analysis and incorporation of technologies into the healthcare system.

**Methods:**

SISALRIL, an autonomous entity created by Law 87-01, evaluates and updates the impact of the Basic Health Plan. Since 2020, it has been part of RedETSA, the Health Technology Assessment Network of the Americas, and leads EVTESA, which was institutionalized in 2022 through Resolution No. 0010-2022. EVTESA ensures evidence-based decisions for including technologies in the Dominican Family Health Insurance, promoting transparency, participation, and financial sustainability, while optimizing resources and improving healthcare quality. EVTESA represents a key methodological framework for evaluating and prioritizing healthcare technologies in the Dominican Republic, transforming the evaluation of technology inclusion and exclusion, and modernizing the historical approach to analyzing benefits for the population.

**Results:**

The implementation of EVTESA has established a structured framework with key documents, such as Resolution No. 0010-2022, which institutionalizes the process, and the methodological manuals for prioritizing and evaluating health technologies. The process includes the reception and verification of requests, evaluation using standardized methodologies, report approval after consensus with medical societies and patients, and decision-making by the National Health Insurance Council regarding the inclusion of technologies in the Dominican Social Security System. The prior analysis for the inclusion of coverage in the plan has strengthened coverage policies, resulting in the allocation of benefits to populations where the greatest clinical impact is achieved and healthcare spending is used more efficiently.

**Conclusions:**

EVTESA marks a pivotal advancement in the Dominican Republic’s social security system by optimizing resources and ensuring access to effective health technologies. This evidence-based, equitable model enhances healthcare quality while promoting sustainability, transparency, and public trust. It also serves as a benchmark for other developing countries, demonstrating how systematic, transparent decision-making can strengthen health systems.

